# High-yield production of human Dicer by transfection of human HEK293-EBNA1 cells grown in suspension

**DOI:** 10.1186/s12896-018-0485-3

**Published:** 2018-12-06

**Authors:** Jonathan Bouvette, Dursun Nizam Korkut, Aurélien Fouillen, Soumiya Amellah, Antonio Nanci, Yves Durocher, James G. Omichinski, Pascale Legault

**Affiliations:** 1 Département de Biochimie et Médecine Moléculaire, Université de Montréal, C.P. 6128, Succursale Centre-Ville, Montréal, H3C 3J7 QC Canada; 2 Département de Stomatologie, Université de Montréal, C.P. 6128, Succursale Centre-Ville, Montréal, QC H3C 3J7 Canada; 3 Human Health Therapeutics Research Centre, National Research Council Canada, Montréal, QC H4P 2R2 Canada

**Keywords:** Human HEK293-EBNA1 cells, Mammalian cell suspension culture, Dicer expression, Dicer purification, Pre-miRNA, Pre-let-7, Dicer binding, Dicer cleavage assay

## Abstract

**Background:**

Dicer is a 219-kDa protein that plays key roles in gene regulation, particularly as the ribonuclease III enzyme responsible for cleaving precursor miRNA substrates. Its enzymatic activity is highly regulated by protein factors, and this regulation can impact on the levels of miRNAs and modulate the behavior of a cell. To better understand the underlying mechanisms of regulation, detailed enzymatic and structural characterization of Dicer are needed. However, these types of studies generally require several milligrams of recombinant protein, and efficient preparation of such quantities of pure human Dicer remains a challenge. To prepare large quantities of human Dicer, we have optimized transfection in HEK293-6E cells grown in suspension and streamlined a purification procedure.

**Results:**

Transfection conditions were first optimized to achieve expression levels between 10 and 18 mg of recombinant Dicer per liter of culture. A three-step purification protocol was then developed that yields 4–9 mg of purified Dicer per liter of culture in a single day. From SEC-MALS/RI analysis and negative stain TEM, we confirmed that the purified protein is monomerically pure ( ≥ 98%) and folds with the characteristic L-shape geometry. Using an electrophoretic mobility shift assay, a dissociation constant (*K*_*d*_) of 5 nM was measured for Dicer binding to pre-let-7a-1, in agreement with previous reports. However, when probing the cleavage activity of Dicer for pre-let-7a-1, we measured *k*_*cat*_ (7.2 ± 0.5 min^− 1^) and *K*_*M*_ (1.2 ± 0.3 μM) values that are much higher than previously reported due to experimental conditions that better respect the steady-state assumption.

**Conclusions:**

The expression and purification protocols described here provide high yields of monomerically pure and active human Dicer. Cleavage studies of a pre-let-7 substrate with this purified Dicer reveal higher *k*_*cat*_ and *K*_*M*_ values than previously reported and support the current view that conformational changes are associated with substrate binding. Large quantities of highly pure Dicer will be valuable for future biochemical, biophysical and structural investigations of this key protein of the miRNA pathway.

**Electronic supplementary material:**

The online version of this article (10.1186/s12896-018-0485-3) contains supplementary material, which is available to authorized users.

## Background

Dicer is a multi-functional protein that plays a critical role in regulating several fundamental cellular processes, including RNA interference, genome integrity, development, and antiviral immunity (for recent reviews see [1, 2]). It is a type III endoribonuclease (RNAse) that contributes to RNA interference by processing specific hairpin structures and long double-stranded RNAs into microRNAs (miRNAs) and small interfering RNAs (siRNAs), respectively. In the miRNA pathway, Dicer is responsible for cleaving the precursor-miRNA (pre-miRNA) into a ~ 22-nucleotide (nt) duplex, from which one strand will be loaded into the RNA-induced silencing complex (RISC) to repress mRNA translation. Currently, it is thought that Dicer produces almost all miRNAs in human cells (~ 2000), which together control over 60% of protein-coding genes [3–7]. The pre-miRNA processing activity of Dicer is known to be highly regulated, and this likely contributes to precisely control miRNA levels and thereby determine cell behavior and cell fate. The cleavage activity is regulated by protein cofactors, such as the **T**AR **R**NA **B**inding **P**rotein (TRBP) and the **P**rotein **ACT**ivator of the interferon-induced protein kinase (PACT), as well as by several RNA-binding proteins known to act either as activators or inhibitors (reviewed in [8]). Moreover, high-throughput studies have identified additional non-coding RNAs that are likely processed by Dicer [9] as well as several pre-miRNA binding proteins that may regulate its cleavage activity [10–12]. At this time, there is a need to integrate the available data into a coherent and detailed mechanistic understanding of Dicer’s activities and regulation via in vitro biochemical, biophysical and structural studies. However, for such research investigations to be carried out effectively, large quantities of highly pure and active recombinant proteins are needed.

Although bacterial overexpression systems generally provide a simple and fast method to obtain significant amount of recombinant proteins, it has not proven practical for human Dicer (219 kDa). Eukaryotic expression systems offer an important alternative for expression of such a large eukaryotic protein because they allow for proper folding and post-translational modifications (reviewed in [13]). Over the past 15 years, purification of recombinant human Dicer following expression in insect cells (Sf9) infected by baculovirus has been achieved with yields up to 0.5–1 mg/L culture [14–16]. This has allowed for the in vitro characterization of human Dicer’s enzymatic activity [14, 15, 17–25] and provided the first descriptions of its three-dimensional structure by cryo-electron microscopy (cryo-EM) at 20–30 Å-resolution [26–28]. Subsequently, the production method in insect cells was improved by systematic optimization of the overexpression and purification steps to yield milligram amounts (3–4 mg/L culture) of highly pure *Drosophila melanogaster* Dicer-2 (dmDicer-2) [29], which was used for its structure determination by cryo-EM at 7-Å resolution. More recently, expression in HEK293 cells grown in suspension was reported as part of a cryo-EM study of human Dicer that allowed structural reconstruction at 4.4-Å resolution [30]. This study provided unprecedented details into Dicer’s domain organization as well as its interaction with TRBP and a pre-miRNA substrate. However, the optimization of Dicer production and the yields obtained were not reported. Therefore, it is possible that large-scale expression from mammalian cells grown in suspension could be optimized to provide pure protein at higher yields than currently reported. Such a procedure could be useful for future biochemical and structural investigations as there is still no X-ray or cryo-EM structure of human Dicer at atomic resolution and there are many questions that remain about its mechanism and regulation by protein factors.

Here, we describe an efficient and fast procedure to obtain milligram quantities of pure, monomeric and stable recombinant human Dicer. To overcome the challenge of Dicer over-expression, we optimized transfection and expression in an HEK293-EBNA1 cell line. This allowed for purification of up to 9 mg of Dicer per liter of culture. A purification protocol was designed to minimize sample handling as well as to provide maximal recovery, purity and enzymatic activity. Although we noticed that human Dicer forms multimers at high concentration, our final three-step purification yields highly-pure monomeric Dicer. The structure and activity of the purified Dicer were assessed by size-exclusion chromatography coupled to multi-angle light-scattering and refractive index (SEC-MALS/RI), negative stain transmission electron microscopy (TEM), binding assay and steady-state kinetics. Our kinetic analysis for Dicer cleavage of the pre-let-7a-1 substrate were performed under strict steady state conditions and reveals *k*_*cat*_ and *K*_*M*_ values that are much higher than previously reported. Overall, this new method will facilitate future biochemical, biophysical and structural characterization of Dicer, including its interaction with RNA substrates and protein regulators.

## Results

### Transient transfection of recombinant human Dicer

**Fig. 1:**
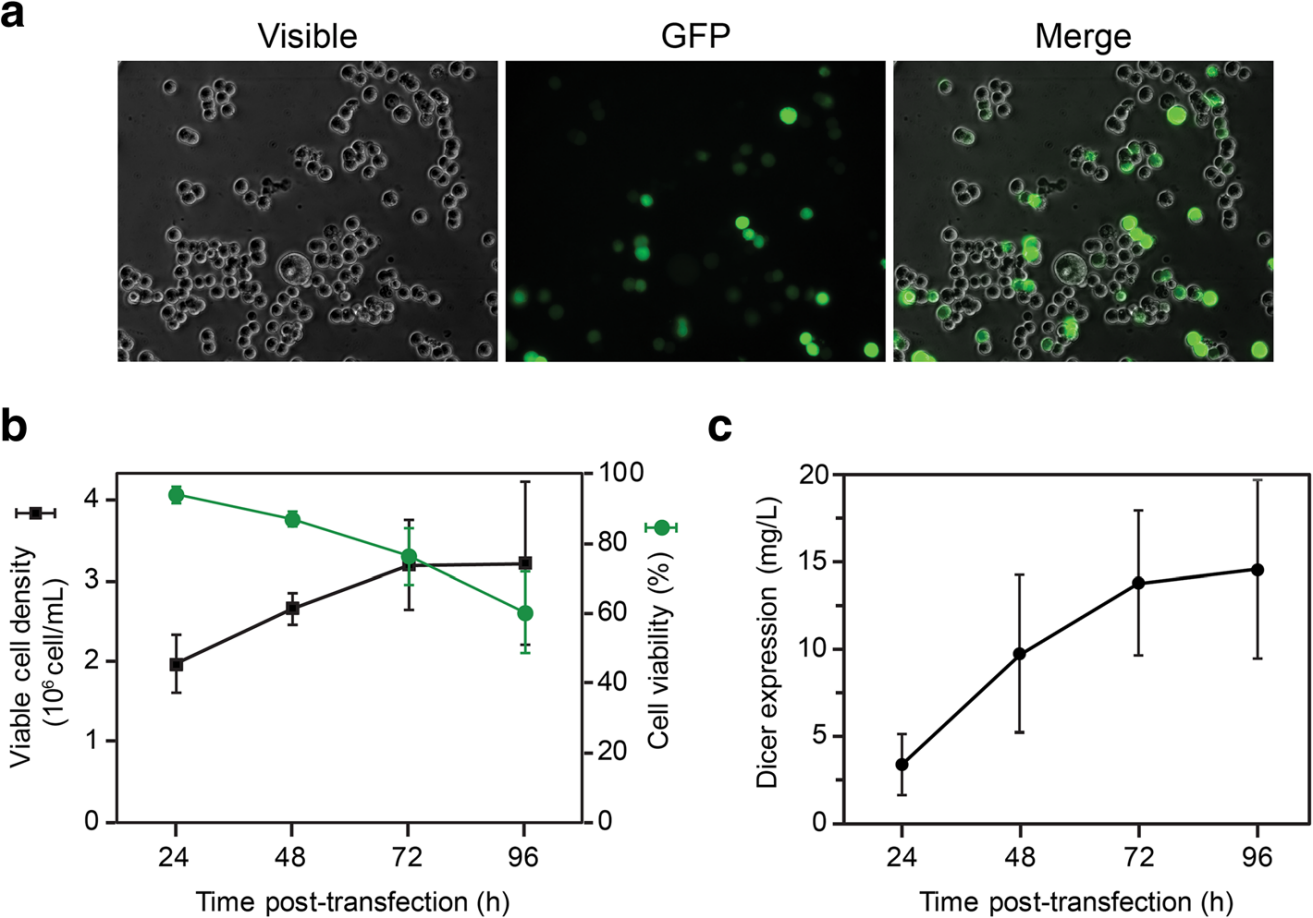
Transfection of small suspension cultures with Dicer-expressing plasmid. 293-6E cells were transfected with PEI:pTT5-DNA complexes at a 2:1 mass ratio and monitored after transfection. (**a**) Transfection efficiency at 48 hpt. A total of 36 ± 7% of the cells are expressing GFP (*n* = 4). (**b**) Cell density and viability (*n* = 4). (**c**) Expression of cytoplasmic Dicer. Dicer quantification was derived from Western blot analysis using a standard curve of pure recombinant Dicer (*n* = 4). Transfections in (**b**) and (**c**) were performed in 20-mL cultures grown in 125-mL Erlenmeyer flasks and the results are means ± standard deviation values of replicates (n) from independent transfections experiments

To achieve high-level expression of recombinant human Dicer, we relied on transient transfection of a suspension-grown HEK-293 cell line expressing the Epstein-Barr virus antigen-1 (HEK293-EBNA1 or 293-6E**)** with a pTT5 expression vector carrying a CMV promoter and the Epstein-Barr virus origin of replication *oriP* [31–33]. This mammalian expression system has been engineered for maximal expression of recombinant His-tagged proteins in serum-free media [34]. Linear polyethyleninime (PEI) was selected as an efficient and cost-effective transfection reagent. For optimization of transfection conditions, small-scale suspension cultures were grown in serum-free media, either as 2-mL cultures in 6-well plates or 20-mL cultures in 20-mL disposable Erlenmeyer flasks. In initial experiments where different quantities of plasmid DNA (1, 1.5 and 2 mg per L of culture) and PEI:DNA ratios (2:1 or 3:1) were tested, we found that optimal transfection efficiency could be reached when transfecting cells diluted to 0.8 × 10^6^ cells/mL 24 h in advance using 1 mg of Dicer-expressing pTT5 plasmid with 2 mg linear PEI per L of culture. To estimate the transfection efficiency, we replaced 5% of the total transfected plasmid by a GFP-expressing plasmid and used fluorescence microscopy to count the percentage of GFP-expressing cells. Transfection efficiencies of 30–40% were typically reached at 48 h post transfection (hpt) (Fig. 1a). Such transfection efficiencies are considered very good especially when taking into account that they are underestimated compared to a transfection using 100% GFP-expressing plasmid [35]. The cell cultures were monitored up to 96 hpt to assess cell viability and viable cell density as well as the intracellular expression of recombinant Dicer. It was found that cell viability decreases below 75% beyond 72 hpt (Fig. 1b, green), whereas viable cell density is maintained at 3.2 × 10^6^ cell/mL (Fig. 1b, black) and Dicer expression levels are not significantly changed (Fig. 1c). Similar results were obtained with large-scale transfections of up to 1 L. Thus, the optimum time for collecting the cells for protein purification was determined to be 72 hpt, taking into consideration that additional time may lead to protein modifications that would adversely affect its activity. At 72 hpt, volumetric expression of the intracellular Dicer protein was 14 ± 4 mg per L of culture on average (Fig. 1c).

### Purification of recombinant human Dicer

**Fig. 2:**
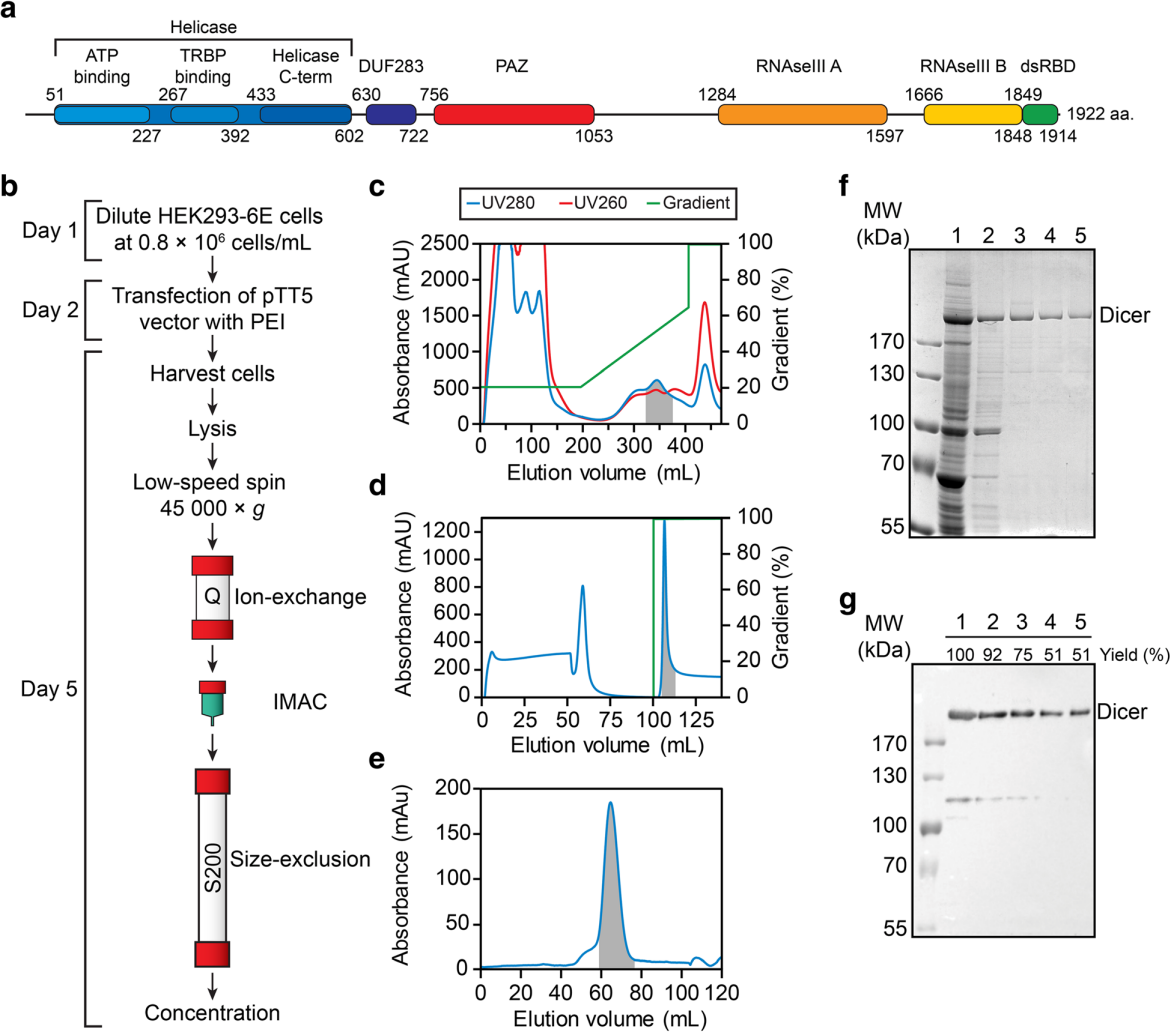
Dicer expression and purification. (**a**) Domain architecture of Dicer. Domains were positioned using InterPro [56] and refined using available structural data [30, 57–59]. (**b**) Flowchart of the Dicer expression and purification protocol. (Day 1) Cells are passaged at 0.8 × 10^6^ cells/mL and incubated 24 h before transfection. (Day 2) The transfection mix is prepared and added to the cell culture, which is incubated for 72 h (37 °C, 5% CO_2_) with shaking (100 RPM). (Day 5) Cells are harvested by centrifugation at 200×g and rinsed 3 times in cold PBS. After lysis, the cytoplasmic fraction is clarified by centrifugation, filtered and loaded on a 60-mL Q Sepharose Fast Flow column for ion-exchange chromatography. Fractions containing Dicer are pooled and loaded directly on a 5-mL HisTrap HP column for purification by immobilized metal affinity chromatography (IMAC). The Dicer-containing fractions are loaded directly on a 120-mL Superdex 200 column for purification by size-exclusion, and the fractions containing homogeneous Dicer are concentrated and stored at − 80 °C. (**c**-**e**) Typical chromatograms from the (**c**) ion-exchange, (**d**) affinity and (**e**) size-exclusion purifications, showing the UV absorbance at 280 nM and 260 nM along with the gradient trace. The selected fractions are highlighted by the grey area. (**f**-**g**) SDS-PAGE summary of the purification viewed by (**f**) Coomassie stain and (**g**) Western blot. Each lane is loaded proportionally to reflect the yield at each step (*Lane 1*: clarified lysate; *Lane 2*: ion-exchange fraction pool; *Lane 3*: affinity fraction pool; *Lane 4*: size-exclusion fraction pool; and *Lane 5*: concentrated protein). Yields were quantified from Western blot analysis, with loaded quantities of Dicer being in the linear range of detection

An efficient multi-step purification protocol was developed to maximize purification yields (Fig. 2b). While optimizing this protocol, we noticed that Dicer precipitated either at high concentration or when in contact with metallic surfaces such as stainless steel needles, FPLC titanium alloy pump heads and the non-reactive Hastelloy sample cell of the isothermal titration calorimeter. Thus, the final purification protocol was optimized to avoid protein contact with metallic surfaces and minimize sample handling that could lead to protein aggregation and precipitation.

Large-scale expression of His-tagged Dicer (400 mL to 1 L) was typically achieved in a 2.8-L glass Fernbach flask and harvested 72 hpt. For cell lysis, the washed cell pellet was resuspended in a low salt buffer containing 0.1% NP-40, allowing for lysis of the plasma membrane while leaving the nuclei intact. Nuclei and cell debris were then removed by centrifugation and filtering of the resulting supernatant. The first step of purification consisted of an anion-exchange chromatography (Q Sepharose Fast Flow), which helped remove protein and nucleic acid contaminants (Fig. 2c). The addition of 10% glycerol in the elution buffers was found critical to purify Dicer free of contamination with Hsp70 (~ 70-kDa intense band in lane 1 of Fig. 2f), which is one of the most abundant protein in the HEK-293 cell line [36]. The Dicer-containing fractions were pooled and directly loaded on a HisTrap HP column for purification by immobilized metal affinity chromatography (IMAC; Fig. 2d). This step, which was carried out in the presence of 0.5 M NaCl and in the absence of glycerol, allowed for the separation of Dicer from the remaining contaminant proteins (Fig. 2f). The Dicer fractions were then loaded on a size-exclusion column (SEC) to isolate the major monomeric population from multimeric contaminants. When this monomeric population of Dicer was concentrated by ultrafiltration under standard conditions (e.g. 50 mM Tris pH 8.2, 10 mM NaCl/KCl 24:1, 0.5 mM MgCl_2_ and 0.5 mM TCEP), it resulted in a significant loss of protein via precipitation. In addition, when Dicer was concentrated prior to SEC purification, substantial amounts of Dicer multimers were detected in the SEC elution profile. This led us to identify conditions that would allow for maximum protein recovery following concentration by ultrafiltration. We found that addition of sucrose and the non-ionic detergent *n*-dodecyl-β-d-maltoside (DDM) helped stabilize the monomeric form. Thus, these reagents were added to the mobile phase during SEC (Fig. 2e, f), and fractions containing monomeric Dicer could be readily concentrated at up to 22.5 μM (5 mg/mL) without precipitation. Analysis of the purified protein by SDS-PAGE gels stained with Coomassie Blue shows greater than 90% purity (Fig. 2f), whereas Western blot analysis indicate that ~ 50% of the protein is recovered from the cytoplasmic fraction (Fig. 2g). Yields of 4–9 mg of human Dicer per liter of culture were typically obtained from large-scale preparations.

### Conformational characterization of the purified Dicer

**Fig. 3:**
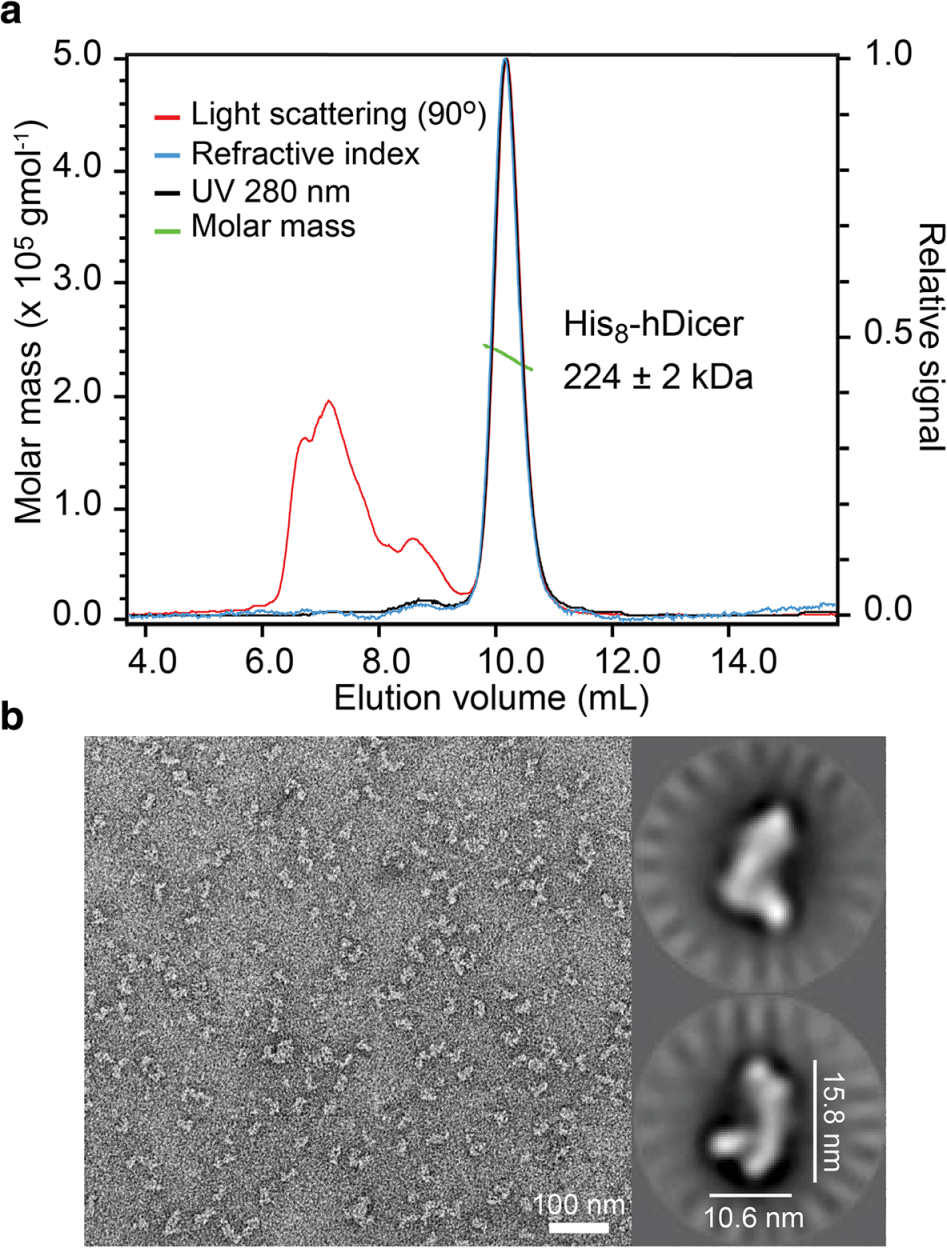
Conformational characterization of purified Dicer. (**a**) SEC-MALS/RI analysis of purified WT Dicer stored in sucrose/DDM-free storage buffer. The relative signal of light scattering (red), refractive index (blue), and UV absorbance at 280 nm (black) are represented by solid lines. The molar mass distribution is shown with green dots. A small amount of aggregated protein can be seen mainly from the light scattering signal. Peak integration of the UV absorbance trace shows that the main peak contains ≥98% of the eluted protein. The molar mass calculated from MALS analysis was normalized against BSA to give an average molecular weight of 224 ± 2 kDa with a polydispersity index of 1.02 ± 0.01 over the entire eluted peak. (**b**) Negative stain TEM. *Left panel* Typical grid imaging of negatively-stained WT Dicer. *Right panels* Main 2D class averages of WT Dicer

Size-exclusion chromatography coupled to multi-angle light-scattering and refractive index (SEC-MALS/RI) is a powerful tool to characterize purified biomolecules in solution; it allows accurate molecular weight determination as well as the determination of the oligomeric state in solution. In the SEC-MALS/RI experiments performed here, SEC served only as a separation step, whereas the LS and RI detectors were used to calculate the molar mass of Dicer after purification and concentration in a sucrose/DDM-free buffer (Fig. 3a). Although a small percentage of multimeric protein appears to be present in the void volume of the SEC (elution volume ~ 8 mL), the purified Dicer elutes predominantly as a single peak that corresponds to ≥98% of the total protein in the injected sample. The derived molecular weight for this peak based on light-scattering data is 224 ± 2 kDa, which essentially matches the theoretical molecular weight of the His-tagged monomeric protein (221 kDa). The polydispersity index of 1.02 ± 0.01 over the entire eluted peak indicates that the main form of purified Dicer is monodisperse, i.e. homogeneous with respect to molar mass. Moreover, SEC-MALS/RI analysis of a purified Dicer sample stored for 6 months at − 80 °C in sucrose/DDM-containing buffer shows that the purified protein remains almost exclusively monomeric ( ≥ 94%), indicating that the protein is intact after long-term storage (Additional file 1: Figure S1).

Negative stain transmission electron microscopy (TEM) is an efficient tool to evaluate the quality of a protein sample, providing information on the homogeneity and the three-dimensional shape of the biomolecule. TEM micrographs of negative stain preparation of the purified Dicer using uranyl formate illustrate particles of roughly equal size throughout the field of view, providing additional evidence of the homogeneity of the purified sample (Fig. 3b). Model-free 2D class averages allowed us to observe the characteristic L-shape architecture of Dicer, with two main unique subpopulations representing different forms of the enzyme, in agreement with previous studies [27, 30, 37]. Taken together, the SEC-MALS/RI and negative stain TEM studies validate the monomeric L-shape architecture of the purified Dicer and indicate that the conformational properties of the enzyme are preserved after long-term storage.

### Binding and cleavage of pre-let-7a-1 by Dicer

**Fig. 4:**
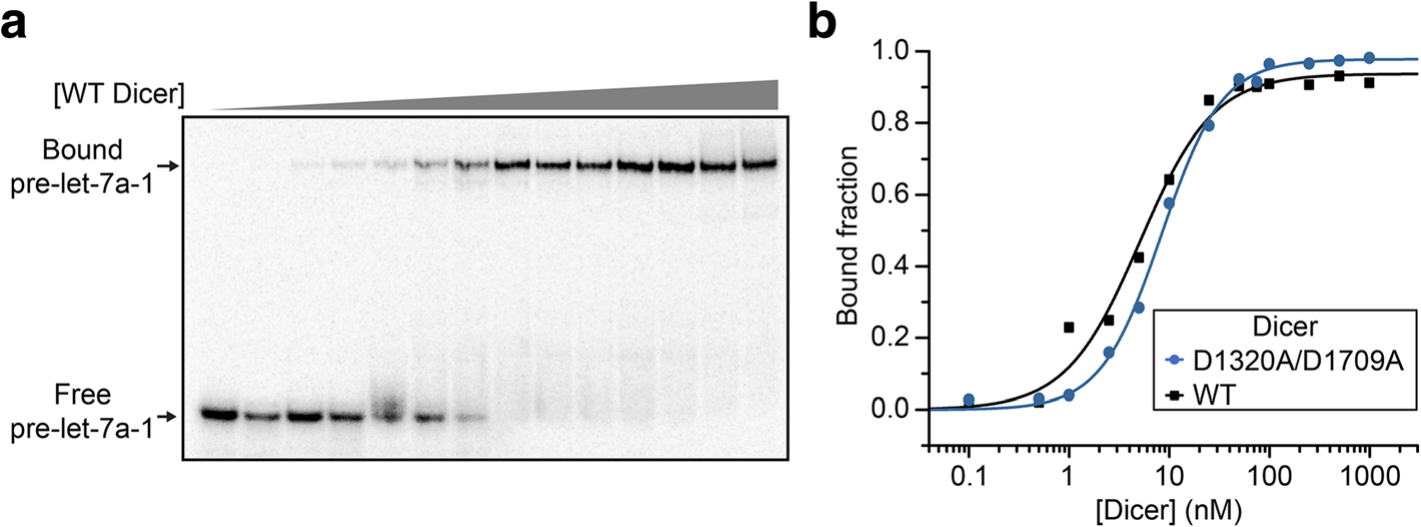
Binding studies of Dicer to pre-let-7a-1. (**a**) Typical EMSA performed with 10 pM of 5′-[^32^P]-labeled pre-let-7a-1 and increasing concentrations of the D1320A/D1709A Dicer variant (0, 0.1, 0.5, 1, 2.5, 5, 10, 25, 50, 75, 100, 250, 500 and 1000 nM). (**b**) Typical binding curves of D1320A/D1709A Dicer and WT Dicer to pre-let-7a-1. The data shown here were fitted to the Hill equation to obtain *K*_*d*_ values of 5.1 ± 0.6 nM (WT Dicer) and 8.4 ± 0.4 nM (D1320A/D1709A Dicer) with Hill coefficient of 1.2 ± 0.1 and 1.4 ± 0.1, respectively. Averages from at least three independent experiments yield *K*_*d*_ values of 5 ± 1 nM (WT Dicer) and 9 ± 1 nM (D1320A/D1709A Dicer) with Hill coefficient of 1.3 ± 0.2 and 1.4 ± 0.3, respectively

Dicer is known to bind pre-miRNA substrates and then cleave at two specific phosphodiester bonds to yield miRNA-5p/3p species. To evaluate the RNA binding and cleavage activity of the purified recombinant Dicer, we performed binding and kinetic studies using the pre-let-7a-1 substrate, one of the most commonly used pre-miRNA substrate for Dicer characterization. Binding studies were conducted via electrophoretic mobility shift assay (EMSA) using ^32^P-labeled pre-let-7a-1 and increasing concentration of Dicer, either wild-type (WT) Dicer supplemented with 0.5 mM EDTA to prevent cleavage or with the catalytically-inactive D1320/1709A variant (Fig. 4). When fitting the binding data to the Hill equation, dissociation constants (*K*_*d*_) of 5 ± 1 nM (WT Dicer) and 9 ± 1 nM (D1320A/D1709A Dicer) were obtained with Hill coefficient of 1.3 ± 0.2 and 1.4 ± 0.3, respectively.

**Fig. 5:**
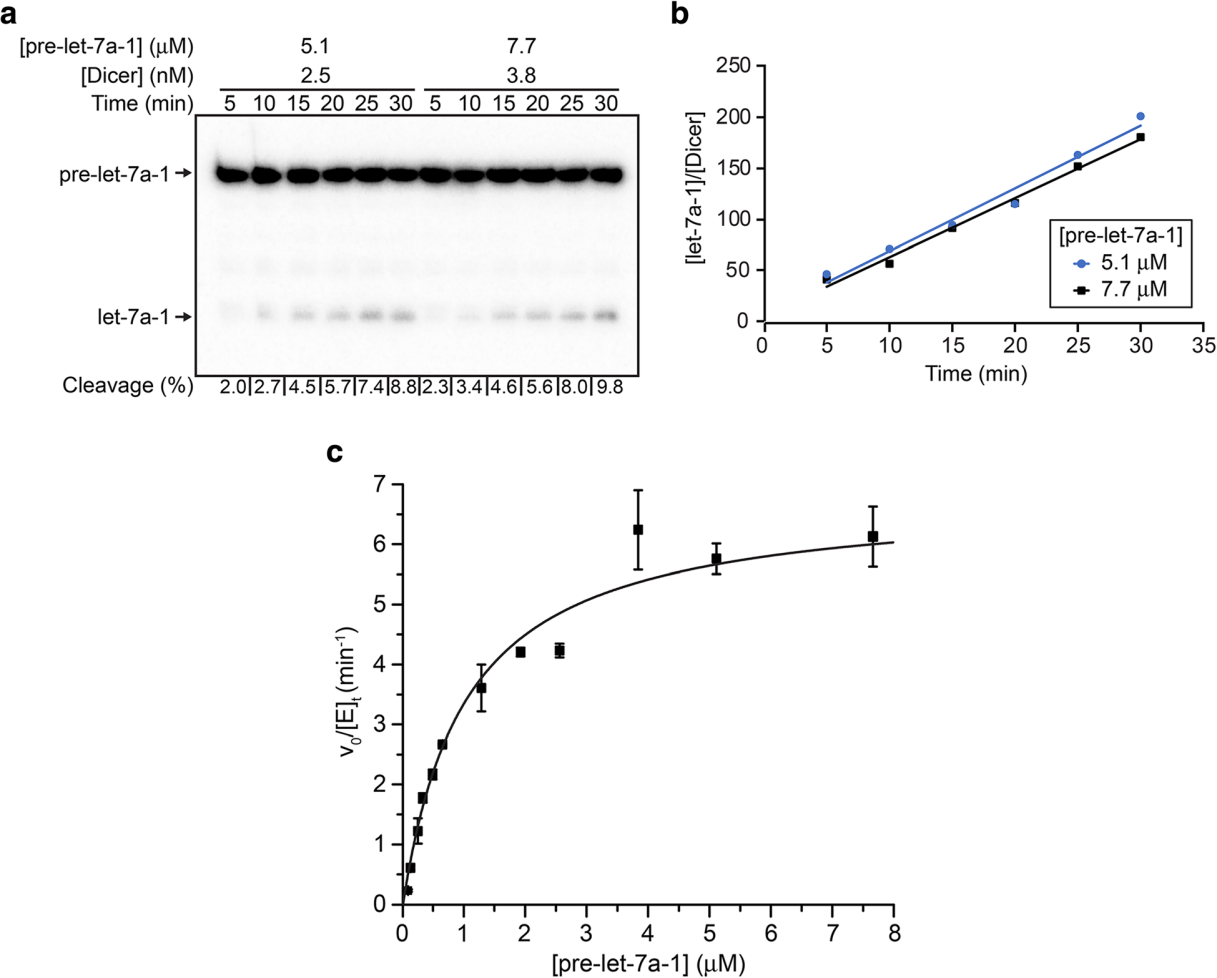
Steady-state kinetics for cleavage of pre-let-7a-1 by Dicer. (**a**) Typical Dicer cleavage reaction of 5′-[^32^P]-labeled pre-let-7a-1 with Dicer analyzed by denaturing gel electrophoresis. Reactions were carried for 30 min in conditions that allowed less than 10% of total substrate cleavage. (**b**) Normalized product formation ([let-7a-1]/[Dicer]) as a function of time for cleavage data shown in (a). The turnover frequency, expressed as *v*_*o*_/[E]_t_, was calculated by linear regression of the slope (5.8 ± 0.3 min^− 1^ and 6.1 ± 0.5 min^− 1^ for pre-let-7a-1 substrate concentration of 5.1 μM and 7.7 μM, respectively). (**c**) Typical steady-state kinetic analysis showing the dependence of the turnover frequency on substrate concentration (0.08 to 7.68 μM). The data shown here were fitted to the Michealis-Menten equation to yield *k*_*cat*_ of 6.8 ± 0.8 min^− 1^ and *K*_*M*_ of 1.0 ± 0.2 μM. Averages from two independent experiments yield *k*_*cat*_ of 7.2 ± 0.5 min^− 1^ and *K*_*M*_ of 1.2 ± 0.3 μM

To characterize the cleavage activity of the purified Dicer enzyme, we determined the steady-state kinetic parameters *k*_*cat*_ and *K*_*M*_ under multiple turnover conditions. To ensure that the initial cleavage rates were measured under steady state conditions, the enzyme concentration ([E]; 0.35 nM to 3.75 nM) was varied along with the initial substrate concentration ([S]; 0.08 μM to 7.68 μM) to maintain [S]/[E] ≥ 200, and time points were collected after at least one turnover of the enzyme pool at up to 10% substrate cleavage, as previously reported for steady-state kinetic studies of *E. coli* RNase III [38]. For each substrate concentration, the cleavage reaction of ^32^P-labeled pre-let-7a-1 was monitored by denaturing gel electrophoresis to quantify the percentage of cleavage (Fig. 5a). From this data, the concentration of product normalized against enzyme concentration ([let-7a-1]/[Dicer]) was plotted as a function of time, and the slope of the resulting time course was fitted by linear regression to derive the turnover frequency, expressed as *v*_*o*_/[E]_t_ (Fig. 5b). By plotting the dependence of *v*_*o*_/[E]_t_ versus [S] and fitting to the Michaelis-Menten equation, a *k*_*cat*_ value of 7.2 ± 0.5 min^− 1^ and a *K*_*M*_ value of 1.2 ± 0.3 μM were determined for cleavage of pre-let-7a-1 by Dicer (Fig. 5c).

## Discussion

By optimizing transfection conditions, we have achieved high-level expression of recombinant human Dicer using a 293-6E cell expression system. In addition, a fast and efficient purification procedure was developed that yields up to 9 mg of pure, monomeric and stable recombinant human Dicer per liter of culture. To our knowledge this yield is higher than previously reported for human Dicer, and it may be possible to obtain even higher yields by further optimizing transfection conditions, expressing Dicer as a secreted protein, and/or developing an efficient stable transfection clone [31, 35, 39, 40]. The reported purification protocol was designed to prevent protein aggregation and precipitation, since we observed that Dicer tends to form multimers and precipitate at high-concentration or when it contacts metallic surfaces. Nevertheless, the purified protein was shown to be monomerically pure and highly active.

Although affinity purification is often considered the best choice for the first purification step of a recombinant protein from a crude mixture, we selected an alternate approach to minimize sample handling that could lead to aggregation and precipitation, such as protein dialysis, desalting, and concentration. Following cell lysis in low-salt buffer, the cytoplasmic fraction was first loaded on an anion-exchange column. The large volume and high salt concentration of the elution fractions from this first purification step is compatible with direct loading onto the HisTrap affinity column. Subsequently, the small elution volume from the second purification step allowed for a single injection on the preparative size-exclusion column without any additional manipulations. Overall, this strategy allows for multi-step purification of milligram amounts of recombinant Dicer in a fast and efficient manner. In fact, the entire purification procedure can be conveniently completed in a single work day.

It is important to note that the last step of purification can be performed either in the presence or absence of reagents (sucrose and DDM) that were found to prevent aggregation and precipitation at high protein concentration. These reagents are not required if the concentration of the protein eluted from the size-exclusion column, generally between 0.5–1 μM, is sufficient for subsequent applications. However, if higher protein concentrations are required and/or long-term storage is desirable, these reagents should be included in the buffer used for size-exclusion chromatography and final protein concentration. The SEC-MALS results indicate that the purified Dicer remains monomeric over six months of storage in sucrose/DDM-supplemented buffer. In agreement with this observation, similar thermodynamic and kinetic parameters (within two-fold) were measured over a period of nine months.

In binding studies, we determined *K*_*d*_ values of 5 ± 1 nM (WT Dicer) and 9 ± 1 nM (D1320A/D1709A Dicer) between Dicer and pre-let-7a-1. These values are similar to previously reported *K*_*d*_ values of 1.8–39 nM obtained for the binding of WT Dicer to pre-let-7a-1 under similar conditions [18, 20, 41, 42]. Moreover, our studies confirm observations with other RNAseIII enzymes that the amino acid substitutions that disrupt magnesium binding in the RNaseIIIa and RNAseIIIb domains do not affect substrate binding to Dicer [43].

From kinetic studies of pre-let-7a-1 cleavage by Dicer, we obtained a *k*_*cat*_ value of 7.2 ± 0.5 min^− 1^ and a *K*_*M*_ value of 1.2 ± 0.3 μM (*k*_*cat*_/*K*_*M*_ = 6 ± 1 min^− 1^ μM^− 1^). These steady-state kinetic parameters are much higher than those derived from previous cleavage studies of the same pre-miRNA substrate (25–375 nM) by Dicer (5 nM): *k*_*cat*_ = 0.45 min^− 1^ and *K*_*M*_ = 20–30 nM (*k*_*cat*_/*K*_*M*_ ~ 18 min^− 1^ μM^− 1^) [20]. Notably, we found similar values (*k*_*cat*_ = 0.8 ± 0.1 min^− 1^ and *K*_*M*_ = 66 nM ± 2 nM; *k*_*cat*_/*K*_*M*_ = 12 ± 2 min^− 1^ μM^− 1^) when performing kinetic experiments under similar conditions (5 nM Dicer and 2.5–1000 nM substrate; Additional file 1: Figure S2). However, the resulting curve did not fit a hyperbolic model, which is understandable given that the following condition, [S] ≫ [E], was not respected for every substrate concentration and, thus, did not agree with the steady-state assumption [44]. By selecting experimental conditions that ensured the validity of the steady-state assumption ([S]/[E] ≥ 200), much higher *k*_*cat*_ and *K*_*M*_ values are obtained, even though the *k*_*cat*_/*K*_*M*_ is not substantially affected (Additional file 1: Table S1).

These updated steady-state kinetic values provide insights into the kinetic mechanism of pre-miRNA cleavage by Dicer. Given the *k*_*cat*_/*K*_*M*_ of ~ 10^5^ M^− 1^ s^− 1^ and the very low *k*_*cat*_ value of ~ 0.1 s^− 1^, the rate-limiting step may be product release as observed with other RNAse III enzymes [38, 45] or possibly the slow formation and/or conversion of the enzyme-substrate complex into a catalytically productive conformation. Dicer is a complex and dynamic enzyme that is known to interact with several cellular proteins [46], and there is increasing evidence that conformational changes may be important for its cleavage activity. Free Dicer is known to adopt different conformations, as confirmed here from negative stain TEM studies, and binding of pre-miRNA or double-stranded RNA substrates is linked to structural rearrangements [18, 23, 27, 37]. Furthermore, single-molecule fluorescence studies have provided evidence for Dicer adopting two different binding modes with a pre-let-7a-1 substrate [47]. More recently, cryo-EM reconstructions of a pre-miRNA/Dicer/TRBP complex captured two distinct conformations of a pre-dicing state, from which transition to the dicing state would require repositioning of the pre-miRNA in the enzyme processing center [30]. Thus, additional studies are needed to better define the detailed kinetic mechanism of Dicer and characterize the role that conformational changes play along the cleavage reaction pathway.

## Conclusion

We have achieved large-scale expression of recombinant human Dicer in a human cell line. The expression in 293-6E cells coupled with our streamlined purification protocol yields up to 9 mg of protein per L of cell culture, which is higher than with previously reported protocols. The purification scheme was carefully optimized to minimize aggregation and maximize monomeric purity and stability of the final sample. To achieve these objectives, we minimized sample manipulation which, in turn, reduced the duration of the purification to a single work day. The purified enzyme adopts the typical L-shape architecture and binds a pre-let-7a-1 substrate in the low nM range, as previously observed. Steady-state kinetic studies yielded higher *k*_*cat*_ and *K*_*M*_ values than previously reported due to the use of experimental conditions that respected the steady-state assumption. Together, these studies provide an efficient method for high-yield production of human Dicer as well as revised kinetic parameters for Dicer cleavage that should prove useful for future structural and mechanistic studies of Dicer aimed at better understanding how the catalytic production of miRNAs is achieved and regulated.

## Methods

### Plasmids

For construction of the human Dicer expression vector pTT5-Dicer, a PCR fragment encoding Dicer was first generated from plasmid pFRT/TO/FLAG/HA-DEST DICER [provided as a gift from Thomas Tuschl (Addgene plasmid # 19881)] [48] and inserted between the NotI and HindIII sites of the pTT5SH8Q1 vector to allow for expression of Dicer with a C-terminal StrepTagII/His_8_ tag [34]. The expression vector for the catalytically-inactive Dicer D1320A/D1709A variant was prepared from pTT5-Dicer using the Stratagene QuikChangeII site-directed mutagenesis method. Plasmids were amplified in *Escherichia coli* (*E. coli*) DH5α cells (Invitrogen) grown in LB medium supplemented with 100 mg/L amplicilin and purified using the QIAGEN Plasmid Giga Kit. Plasmids were stored at 4 °C in water at a concentration of 1 mg/mL.

The pUC19-HH-pre-let-7a-1-HDV vector used for in vitro transcription of pre-let-7a-1 was constructed using a pUC19 vector, which allows for synthesis of pre-let-7a-1 flanked by a 5’ Hammerhead ribozyme (HH) and a 3’ Hepatitis delta virus (HDV) ribozyme. Plasmids were amplified in *E. coli* DH5α cells grown in LB medium supplemented with 100 mg/L ampicillin and purified using the QIAGEN Plasmid Maxi Kit. Plasmids were linearized with HindIII (New England Biolabs) and stored as is at − 20 °C at a concentration of 1 mg/mL. All plasmids were verified by DNA sequencing.

### Cell culture and transfection

HEK-293-EBNA1-6E (293-6E) cells were grown in suspension in Freestyle 17 (F17) medium (Invitrogen) supplemented with 4 mM glutamine, 0.1% pluronic acid and 25 μg/mL G418 at 37 °C with 5% CO_2_. Tryptone N1 was added to some of the cell cultures at a weight/volume ratio of 0.5%, but was found to have minimal effect on cultures grown 72 hpt. Cultures were maintained under 2.0 × 10^6^ cells/mL in Erlenmeyer flasks shaken at 100 RPM in a humidified incubator at 37 °C with 5% CO_2_.

Cell cultures were diluted to 0.8 × 10^6^ cells/mL 24 h before transfection. The transfection mix was prepared in 10% of the final culture volume in F17 medium with 10 μg/mL of DNA plasmid and 20 μg/mL of linear PEI 25 kDa (Polysciences Inc.). The mix was agitated by 3 vortexing cycles of 3 s, incubated 15 min at room temperature and incorporated in the culture [32], which was incubated for 72–96 h (37 °C, 5% CO_2_) with shaking (100 RPM).

For optimization of transfection conditions, small-scale cultures were used: 2-mL cultures were grown in 6-well plates (Starstedt #83.1839.500) and 20-mL cultures were grown in 125-mL disposable Erlenmeyer flasks (Corning #431143). For Dicer purification, large-scale cultures of 150 mL to 1 L were grown in thoroughly rinsed and sterilized 1-L glass Erlenmeyer (Corning #49851 L) or 2.8-L Fernbach culture flasks (Corning #44262×L). The incubation time after transfection was fixed to 72 h for large-scale cultures.

At harvest time, cells were first counted and then pelleted by centrifugation at 200×*g* and washed 3 times with at least 5-pellet volumes of cold PBS. The washed cell pellet was either used immediately or frozen in liquid nitrogen and stored at − 80 °C for later use.

### Optimization of transfection conditions

To optimize transfection conditions, small-scale transfections were monitored over a 4-day period by withdrawing a 190-μL aliquot from the culture every 24 h to measure cell density, cell viability and recombinant Dicer expression. From this aliquot, a 10-μL volume was mixed with an equal volume of trypan blue and loaded on a hemocytometer slide to determine the cell density and viability using a Nikon Eclipse TS100 microscope. The remaining 180 μL of cell culture was pelleted, resuspended in 60 μL of lysis buffer (20 mM Tris pH 7.4, 50 mM NaCl, 10% Glycerol, 1 mM EDTA, 1 mM EGTA, 0.1% NP-40 and 0.5 mM TCEP [tris(2-carboxyethyl)phosphine]) and spun for 1 min at 16,000×*g*. The supernatant containing the cytoplasmic fraction was mixed with one volume of 2X Laemmli buffer and stored at − 20 °C. All samples collected during transfection were heated at 95 °C for 2 min, separated on a 7.5% SDS-PAGE and blotted on a nitrocellulose membrane (Bio-Rad) for Western-blot analysis. Dicer was detected using a mouse anti-His tag primary antibody (Medimabs #MM-0165-P), an HRP-conjugated anti-mouse secondary antibody (Cedarlane #115–035-044) and the Western Lightning Plus-ECL substrate (Perkin-Elmer #NEL103E001EA). A ChemiDoc MP (Bio-Rad) Imaging system was used for chemiluminescence detection, and band intensities were quantified using the ImageLab software version 5.2 (Bio-Rad).

Transfection efficiency was monitored by substituting 5% of the total plasmid transfected by a pTT5SH8Q1-GFPq plasmid [49]. After 48 h, live cells were diluted with one volume of Freestyle 17 media supplemented with 20 mM HEPES pH 7.5, and a thin layer of diluted cells was carefully deposited on a Lab-TEK-II chamber slide. The slide was visualized by both phase contrast microscopy and by fluorescence microscopy on a Nikon TE2000U microscope (20× magnification). The ratio of cells expressing GFP over the total cells of at least 200 cells was taken as the transfection efficiency.

### Dicer purification

Dicer-transfected cell pellets were resuspended in 5-pellet volumes of lysis buffer supplemented with one tablet of Roche Complete EDTA-free and incubated on ice for 10 min. The lysate was clarified by centrifugation at 45000×*g* for 20 min at 4 °C. The supernatant was then filtered on a 0.22-μm pore size polyethersulfone (PES) membrane and loaded onto a 60-mL Q Sepharose Fast Flow media packed in a XK 26/20 column (GE Healthcare) equilibrated with Qseph-A buffer (20 mM Tris pH 7.4, 10% glycerol and 0.5 mM TCEP). After loading, the column was washed with 2.5 column volume (CV) of 20% Qseph-B buffer (20 mM Tris pH 7.4, 1 M NaCl, 10% glycerol and 0.5 mM TCEP) and Dicer was eluted using a linear gradient (from 20 to 70% over 4 CV) of Qseph-B. The Dicer-containing fractions were combined and loaded on a 5-mL HisTrap High Performance column (GE Healthcare) equilibrated with His-A buffer (20 mM Tris pH 7.4, 500 mM NaCl, 25 mM imidazole and 0.5 mM TCEP). After loading, the column was washed with 12 CV of His-A buffer and Dicer was eluted with a single isocratic step of 100% His-B buffer (20 mM Tris pH 7.4, 500 mM NaCl, 250 mM imidazole and 0.5 mM TCEP). The fractions containing Dicer were combined and loaded on a preparative Superdex 200 16/600 column (GE Heatlhcare) pre-equilibrated in storage buffer, either sucrose/DDM-free storage buffer (50 mM Tris pH 8.2, 10 mM NaCl/KCl 24:1, 0.5 mM MgCl_2_, and 0.5 mM TCEP) or storage buffer supplemented with 5% Sucrose and 0.3 mM DDM (*n*-dodecyl-β-d-maltoside). When needed, fractions containing monomeric Dicer were pooled and concentrated on a 7-mL Apollo concentrator 150-kDa MWCO (Orbital Biosciences). The concentrated Dicer was aliquoted, flash frozen in liquid nitrogen and stored at − 80 °C for future use. Purity and yield were assessed by 7.5% Coomassie-stained SDS-PAGE and Western blotting of the different fractions collected during purification.

### Synthesis and purification of pre-let-7a-1

The in vitro transcription of human pre-let-7a-1 with a 5’-HH ribozyme tag and a 3’-HDV ribozyme tag was carried out in 40 mM HEPES pH 8.0, 20 mM MgCl_2_, 50 mM DTT, 1 mM spermidine, 0.1% triton X-100, 4 mM of each NTP, 30 μg/mL of linearized pUC19-HH-pre-let-7a-1-HDV plasmid, 0.5 U/mL of RNasin® Plus RNase inhibitor and 30 μg/mL of His-tagged T7 polymerase prepared in house [50]. Since both ribozyme tags were cleaved off during transcription, the pre-let-7a-1 RNA was generated with homogeneous ends directly from the transcription reaction. The RNA was then purified by denaturing gel electrophoresis, as described previously [51]. To convert the purified RNA into an optimal Dicer substrate with 5′-phosphate and 3’-OH ends [52], a phosphorylation reaction was carried out with T4 polynucleotide kinase (NEB #M0201S) according to the manufacturer protocol with either cold ATP or ATP-γ-^32^P. The ^32^P-labeled pre-let-7a-1 was further purified by denaturing gel electrophoresis, extracted from the gel by crush and soak, ethanol precipitated and resuspended in TE pH 7.5 (10 mM Tris pH 7.5 and 1 mM EDTA) [51]. The cold phosphorylated RNA was further purified by HPLC at 65 °C on a DNApac PA-100 9 × 250 mm equilibrated with 30% DNApac-B buffer (12.5 mM Tris pH 7.4, 5 M urea and 0.5 M NaClO_4_) and 70% DNApac-A buffer (12.5 mM Tris pH 7.4 and 5 M urea). The sample was loaded and eluted with a linear gradient (30 to 70%) of DNApac-B buffer. The fraction containing pure pre-let-7a-1 was exchanged into TE pH 7.5 and concentrated using a 3-kDa MWCO Amicon® Ultra-4 Centrifugal Filter Unit (Millipore). Both the ^32^P-labeled and cold pre-let-7a-1 samples were stored at − 20 °C.

### Sec-MALS/RI

Human WT Dicer final purification product was analyzed by SEC-MALS/RI using an AKTA micro FPLC (GE Healthcare) coupled to a multiple-angle light scattering and refractive index system (MALS/RI; Wyatt Dawn HELEOS II and OptiLab T-rEX). A volume of 100 μL (75–80 μg) of purified Dicer was loaded on a Superdex 200 Increase 10/300 (GE Healthcare) column equilibrated in sucrose/DDM-supplemented storage buffer at a flowrate of 0.3–0.5 mL/min. The SEC-MALS/RI data was collected and analysed with ASTRA chromatography software package, version 6.1.6.5 (Wyatt Technology). The MALS detector was normalized using the monomeric and dimeric signals of bovine serum albumin (BSA; Fisher scientific BP1605). For the Dicer sample purified in DDM-free buffer, the molar mass was determined by the dual detection method (RI/LS signals) implemented in the conjugated analysis mode of the ASTRA software, using a refractive index increment (dn/dc) of 0.190 mL/g [53]. A similar method was used for the Dicer sample purified in DDM-containing buffer, however the dual detection method used the UV and LS signals, because the RI signal may be perturbed by DDM-Dicer interactions.

### Negative stain TEM

A 3-μL drop of 50 nM human WT Dicer in 20 mM HEPES, pH 7.5, 150 mM KCl, 3 mM EDTA, 1 mM DTT, and 2.5% glycerol was applied onto a carbon-coated copper grid previously glow-discharged using an ELMO glow discharge system (Cordouan Technologies, France). After 1 min, excess liquid was blotted and stained for 1 min with 1.5% freshly prepared uranyl formate (Electron Microscopy Sciences, PA). Samples were imaged using a FEI Tecnai T12 (Eindhoven, The Netherlands) Transmission Electron Microscope (TEM) equipped with a LaB6 filament and operated at an acceleration voltage of 120 kV. Micrographs were collected at defocus between 1 and 3 μm on a FEI Eagle 4 k × 4 k CCD camera at a magnification of ~ 67,000×. From these micrographs, 125,000 particles were extracted and 2D align using xmipp from Scipion [54]. The particles were classified in a minimum of 2 classes and the 2D volumes were measured using ImageJ [55].

### Dicer binding assay

Binding assays were carried using either WT Dicer or the catalytically-inactive Dicer variant (D1320A/D1709A). Dicer D1320A/D1709A was first diluted in EMSA buffer (100 mM Tris pH 7.6, 100 mM NaCl and 20% glycerol), whereas WT Dicer was diluted in EMSA buffer supplemented with 1 mM EDTA. In parallel, ^32^P-labeled pre-let-7a-1 was refolded by heating at 95 °C for 2 min and snap-cooling at 4 °C for a minimum of 5 min. The RNA was then diluted in 0.1% NP-40 and 4 mM DTT to a concentration of 4 pM. The binding reactions were initiated by adding 10 μL of the RNA sample to 10 μL of the protein samples. Standard binding reactions contained 50 mM Tris pH 7.6, 50 mM NaCl, 10% glycerol, 0.05% NP-40, 2 mM DTT, and 2 pM ^32^P-labeled RNA (0.5 mM EDTA was added for reactions with WT Dicer) with protein concentrations varying between 0.01× and 100× of the estimated *K*_*d*_ value. Reactions were incubated for 30 min at 4 °C and loaded on a 4–15% gradient polyacrylamide gel (37.5:1 acrylamide:bis-acrylamide) in Tris-Glycine buffer (25 mM Tris–Base and 200 mM glycine), which was run for 2 h at 200 V in the cold room (4 °C). Gels were dried, exposed overnight to a storage phosphor screen (Bio-Rad) and visualized with a Personal Molecular Imager (Bio-Rad). Band intensities for the bound (B) and unbound (U) RNA were quantified using the ImageLab software version 5.2 (Bio-Rad). The fraction of bound RNA [F = B/(B + U)] was plotted against protein concentration, and the binding data were fitted to the Hill equation with the OriginPro 8 software (OriginLab). The reported *K*_*d*_ value and its errors represent the average and standard deviation from two independent experiments.

### Dicer cleavage assay

For in vitro Dicer cleavage assays, the RNA and Dicer solutions were prepared separately at twice their final desired concentration in 30 μL of the cleavage reaction buffer (50 mM HEPES pH 7.4, 50 mM NaCl, 5 mM MgCl_2_ and 0.05% NP-40). The RNA solutions, containing 80 pM of ^32^P-labeled pre-let-7a-1 and cold RNA at concentrations ranging from 0.1× to 10× the estimated *K*_*M*_, were heated at 95 °C for 2 min and snap-cooled at 4 °C for a minimum of 5 min to refold the RNA. The concentration of the Dicer solution was adjusted for each pre-let-7a-1 substrate concentration to allow measurement of the initial velocity of the reaction (5–10% substrate cleavage in 30 min) under multiple turnover conditions (substrate/Dicer molar ratio ≥ 200). Both RNA and protein solutions were pre-heated for 5 min at 37 °C, and cleavage reactions were initiated by adding 30 μL of the protein solution to 30 μL of the RNA solution. At specific time points, a 5-μL aliquot of the cleavage reaction was taken, immediately mixed with 5 μL of stop buffer (95% formamide and 50 mM EDTA). For each reaction, 6 time points were taken at less than 10% reaction completion and over at least 2 full turnovers of the enzyme pool to ascertain the possible use of the steady-state assumption. Samples were analyzed by denaturating gel electrophoresis [20% acrylamide:bis-acrylamide (19:1) /7 M urea gel]. The amounts of substrate (S) and product (P) were quantified from radioactive bands as for the binding assay, and the fraction of cleaved product [F = P/ (S + P)] was plotted against time. The resulting time courses were fitted by linear regression and the slope was taken as the initial velocity (*v*_*o*_). Subsequently, the dependence of *v*_*o*_/[E]_t_ on [S] was plotted and fitted to the Michaelis-Menten equation (written as *v*_*o*_/[E]_t_ = *k*_*cat*_[S]/(*K*_*M*_+[S]) with the OriginPro 8 software (OriginLab) to derive *k*_*cat*_ and *K*_*M*_ values. The quality of the fit was obtained from the square of the correlation coefficient (*R*^*2*^), with *R*^*2*^ ≥ 0.97 and by making sure that the distribution of residuals was random around the regression line. The values of *k*_*cat*_ and *K*_*M*_ are reported with experimental errors taken from the average and standard deviations from two independent experiments.

## Additional file

Additional file 1: An additional file named Bouvette_Additional_File1.pdf is available that contain Table S1 as well as Figures S1 and S2. **Table S1.** compares kinetic parameters obtained under conditions that respect or not the steady-state assumption. **Figure S1.** shows SEC-MALS analysis of purified WT Dicer stored in sucrose/DDM-containing storage buffer for 6 months at − 80 °C. **Figure S2.** shows preliminary kinetics studies of pre-let-7a-1 cleavage by Dicer under non-steady-state conditions. (PDF 227 kb)

## Abbreviations

BSA: Bovine serum albumin
CCD: Charge-coupled device
CMV: Cytomegalovirus
CV: Column volume
DDM: *n*-dodecyl-β-d-maltoside
DTT: Dithiothreitol
EBNA1: Epstein-Barr nuclear antigen 1
EM: Electron microscopy
EMSA: Electrophoretic mobility shift assay
ES: Enzyme-substrate
FPLC: Fast protein liquid chromatography
GFP: Green fluorescent protein
HDV: Hepatitis delta virus
HEK293-6E: EBNA1 human embryonic kidney cell line
HH: Hammerhead
Hsp70: Heat-shock protein 70
LS: Light-scattering
MWCO: Molecular weight cut-off
NP-40: Nonyl phenoxypolyethoxylethanol
PACT: Protein activator of the interferon-induced protein kinase
PAGE: Polyacrylamide gel electrophoresis
PBS: Phosphate buffer saline
PEI: Polyethyleninime
PES: Polyethersulfone
RI: Refractive index
RISC: RNA-induced silencing complex
RPM: Revolution per minute
SDS: Sodium dodecyl sulfate
SEC-MALS: Size-exclusion chromatography and multiple-angle light scattering
TCEP: Tris(2-carboxyethyl)phosphine
TEM: Transmission electron microscopy
TRBP: TAR-RNA binding protein
WT: Wild-type

## References

[1] Song MS, Rossi JJ (2017). Molecular mechanisms of dicer: endonuclease and enzymatic activity. Biochem J.

[2] Foulkes WD, Priest JR, Duchaine TF (2014). DICER1: mutations, microRNAs and mechanisms. Nat Rev Cancer.

[3] Kozomara A, Griffiths-Jones S (2014). miRBase: annotating high confidence microRNAs using deep sequencing data. Nucleic Acids Res.

[4] Friedman RC, Farh KK, Burge CB, Bartel DP (2009). Most mammalian mRNAs are conserved targets of microRNAs. Genome Res.

[5] Cheloufi S, Dos Santos CO, Chong MM, Hannon GJ (2010). A dicer-independent miRNA biogenesis pathway that requires ago catalysis. Nature.

[6] Cifuentes D, Xue H, Taylor DW, Patnode H, Mishima Y, Cheloufi S, Ma E, Mane S, Hannon GJ, Lawson ND (2010). A novel miRNA processing pathway independent of dicer requires Argonaute2 catalytic activity. Science.

[7] Havens MA, Reich AA, Duelli DM, Hastings ML (2012). Biogenesis of mammalian microRNAs by a non-canonical processing pathway. Nucleic Acids Res.

[8] Ha M, Kim VN (2014). Regulation of microRNA biogenesis. Nat Rev Mol Cell Biol.

[9] Li Z, Ender C, Meister G, Moore PS, Chang Y, John B (2012). Extensive terminal and asymmetric processing of small RNAs from rRNAs, snoRNAs, snRNAs, and tRNAs. Nucleic Acids Res.

[10] Di Tomasso G, Miller Jenkins LM, Legault P (2016). ARiBo pull-down for riboproteomic studies based on label-free quantitative mass spectrometry. RNA.

[11] Treiber T, Treiber N, Plessmann U, Harlander S, Daiss JL, Eichner N, Lehmann G, Schall K, Urlaub H, Meister G (2017). A compendium of RNA-binding proteins that regulate microRNA biogenesis. Mol Cell.

[12] Nussbacher JK, Yeo GW (2018). Systematic discovery of RNA binding proteins that regulate microRNA levels. Mol Cell.

[13] Geisse S, Gram H, Kleuser B, Kocher HP (1996). Eukaryotic expression systems: a comparison. Protein Expr Purif.

[14] Provost P, Dishart D, Doucet J, Frendewey D, Samuelsson B, Radmark O (2002). Ribonuclease activity and RNA binding of recombinant human dicer. EMBO J.

[15] Zhang H, Kolb FA, Brondani V, Billy E, Filipowicz W (2002). Human dicer preferentially cleaves dsRNAs at their termini without a requirement for ATP. EMBO J.

[16] Myers JW, Jones JT, Meyer T, Ferrell JE (2003). Recombinant dicer efficiently converts large dsRNAs into siRNAs suitable for gene silencing. Nat Biotechnol.

[17] Vermeulen A, Behlen L, Reynolds A, Wolfson A, Marshall WS, Karpilow J, Khvorova A (2005). The contributions of dsRNA structure to dicer specificity and efficiency. RNA.

[18] Ma E, MacRae IJ, Kirsch JF, Doudna JA (2008). Autoinhibition of human dicer by its internal helicase domain. J Mol Biol.

[19] Soifer HS, Sano M, Sakurai K, Chomchan P, Saetrom P, Sherman MA, Collingwood MA, Behlke MA, Rossi JJ (2008). A role for the dicer helicase domain in the processing of thermodynamically unstable hairpin RNAs. Nucleic Acids Res.

[20] Chakravarthy S, Sternberg SH, Kellenberger CA, Doudna JA (2010). Substrate-specific kinetics of dicer-catalyzed RNA processing. J Mol Biol.

[21] Feng Y, Zhang X, Graves P, Zeng Y (2012). A comprehensive analysis of precursor microRNA cleavage by human dicer. RNA.

[22] Lee HY, Zhou K, Smith AM, Noland CL, Doudna JA (2013). Differential roles of human dicer-binding proteins TRBP and PACT in small RNA processing. Nucleic Acids Res.

[23] Liu Z, Wang J, Li G, Wang HW (2015). Structure of precursor microRNA's terminal loop regulates human Dicer's dicing activity by switching DExH/D domain. Protein Cell.

[24] Kurzynska-Kokorniak A, Pokornowska M, Koralewska N, Hoffmann W, Bienkowska-Szewczyk K, Figlerowicz M (2016). Revealing a new activity of the human dicer DUF283 domain in vitro. Sci Rep.

[25] MacRae IJ, Ma E, Zhou M, Robinson CV, Doudna JA (2008). In vitro reconstitution of the human RISC-loading complex. Proc Natl Acad Sci U S A.

[26] Lau PW, Potter CS, Carragher B, MacRae IJ (2009). Structure of the human dicer-TRBP complex by electron microscopy. Structure.

[27] Taylor DW, Ma E, Shigematsu H, Cianfrocco MA, Noland CL, Nagayama K, Nogales E, Doudna JA, Wang HW (2013). Substrate-specific structural rearrangements of human dicer. Nat Struct Mol Biol.

[28] Wang H-W, Noland C, Siridechadilok B, Taylor DW, Ma E, Felderer K, Doudna JA, Nogales E (2009). Structural insights into RNA processing by the human RISC-loading complex. Nat Struct Mol Biol.

[29] Sinha NK, Bass BL (2017). Overexpression and purification of dicer and accessory proteins for biochemical and structural studies. Methods.

[30] Liu Z, Wang J, Cheng H, Ke X, Sun L, Zhang QC, Wang HW (2018). Cryo-EM structure of human dicer and its complexes with a pre-miRNA substrate. Cell.

[31] Durocher Y, Perret S, Kamen A (2002). High-level and high-throughput recombinant protein production by transient transfection of suspension-growing human 293-EBNA1 cells. Nucleic Acids Res.

[32] Tom R, Bisson L, Durocher Y (2008). Transfection of HEK293-EBNA1 cells in suspension with linear PEI for production of recombinant proteins. CSH Protoc..

[33] Tom R, Bisson L, Durocher Y (2008). Culture of HEK293-EBNA1 cells for production of recombinant proteins. CSH Protoc.

[34] Cass B, Pham PL, Kamen A, Durocher Y (2005). Purification of recombinant proteins from mammalian cell culture using a generic double-affinity chromatography scheme. Protein Expr Purif.

[35] L'Abbe D, Bisson L, Gervais C, Grazzini E, Durocher Y (2018). Transient gene expression in suspension HEK293-EBNA1 cells. Methods Mol Biol.

[36] Schirle M, Heurtier MA, Kuster B (2003). Profiling core proteomes of human cell lines by one-dimensional PAGE and liquid chromatography-tandem mass spectrometry. Mol Cell Proteomics.

[37] Lau PW, Guiley KZ, De N, Potter CS, Carragher B, MacRae IJ (2012). The molecular architecture of human dicer. Nat Struct Mol Biol.

[38] Campbell FE, Cassano AG, Anderson VE, Harris ME (2002). Pre-steady-state and stopped-flow fluorescence analysis of Escherichia coli ribonuclease III: insights into mechanism and conformational changes associated with binding and catalysis. J Mol Biol.

[39] Fukumoto Y, Obata Y, Ishibashi K, Tamura N, Kikuchi I, Aoyama K, Hattori Y, Tsuda K, Nakayama Y, Yamaguchi N (2010). Cost-effective gene transfection by DNA compaction at pH 4.0 using acidified, long shelf-life polyethylenimine. Cytotechnology.

[40] Delafosse L, Xu P, Durocher Y (2016). Comparative study of polyethylenimines for transient gene expression in mammalian HEK293 and CHO cells. J Biotechnol.

[41] Lee HY, Doudna JA (2012). TRBP alters human precursor microRNA processing in vitro. RNA.

[42] Ma E, Zhou K, Kidwell MA, Doudna JA (2012). Coordinated activities of human dicer domains in regulatory RNA processing. J Mol Biol.

[43] Li H, Nicholson AW (1996). Defining the enzyme binding domain of a ribonuclease III processing signal. Ethylation interference and hydroxyl radical footprinting using catalytically inactive RNase III mutants. EMBO J.

[44] Leskovac V (2007). Comprehensive enzyme kinetics.

[45] Nicholson AW (2014). Ribonuclease III mechanisms of double-stranded RNA cleavage. Wiley Interdiscip Rev RNA.

[46] Zhang H, Kolb FA, Jaskiewicz L, Westhof E, Filipowicz W (2004). Single processing center models for human dicer and bacterial RNase III. Cell.

[47] Fareh M, Yeom KH, Haagsma AC, Chauhan S, Heo I, Joo C (2016). TRBP ensures efficient dicer processing of precursor microRNA in RNA-crowded environments. Nat Commun.

[48] Landthaler M, Gaidatzis D, Rothballer A, Chen PY, Soll SJ, Dinic L, Ojo T, Hafner M, Zavolan M, Tuschl T (2008). Molecular characterization of human Argonaute-containing ribonucleoprotein complexes and their bound target mRNAs. RNA.

[49] Lindner HA, Fotouhi-Ardakani N, Lytvyn V, Lachance P, Sulea T, Menard R (2005). The papain-like protease from the severe acute respiratory syndrome coronavirus is a deubiquitinating enzyme. J Virol.

[50] Salvail-Lacoste A, Di Tomasso G, Piette BL, Legault P (2013). Affinity purification of T7 RNA transcripts with homogeneous ends using ARiBo and CRISPR tags. RNA.

[51] Bouchard P, Lacroix-Labonte J, Desjardins G, Lampron P, Lisi V, Lemieux S, Major F, Legault P (2008). Role of SLV in SLI substrate recognition by the *Neurospora* VS ribozyme. RNA.

[52] Park JE, Heo I, Tian Y, Simanshu DK, Chang H, Jee D, Patel DJ, Kim VN (2011). Dicer recognizes the 5′ end of RNA for efficient and accurate processing. Nature.

[53] Zhao H, Brown PH, Schuck P (2011). On the distribution of protein refractive index increments. Biophys J.

[54] de la Rosa-Trevin JM, Quintana A, Del Cano L, Zaldivar A, Foche I, Gutierrez J, Gomez-Blanco J, Burguet-Castell J, Cuenca-Alba J, Abrishami V (2016). Scipion: a software framework toward integration, reproducibility and validation in 3D electron microscopy. J Struct Biol.

[55] Schneider CA, Rasband WS, Eliceiri KW (2012). NIH image to ImageJ: 25 years of image analysis. Nat Methods.

[56] Finn RD, Attwood TK, Babbitt PC, Bateman A, Bork P, Bridge AJ, Chang HY, Dosztanyi Z, El-Gebali S, Fraser M (2017). InterPro in 2017-beyond protein family and domain annotations. Nucleic Acids Res.

[57] Qin H, Chen F, Huan X, Machida S, Song J, Yuan YA (2010). Structure of the Arabidopsis thaliana DCL4 DUF283 domain reveals a noncanonical double-stranded RNA-binding fold for protein-protein interaction. RNA.

[58] Tian Y, Simanshu DK, Ma JB, Park JE, Heo I, Kim VN, Patel DJ (2014). A phosphate-binding pocket within the platform-PAZ-connector helix cassette of human dicer. Mol Cell.

[59] Kolakofsky D, Kowalinski E, Cusack S (2012). A structure-based model of RIG-I activation. RNA.

