# Supplementary material for: High-yield production of human Dicer by transfection of human HEK293-EBNA1 cells grown in suspension
Source: BMC Biotechnol. 2018 Dec 6;18:76. doi: 10.1186/s12896-018-0485-3 (PMC6282390; doi:10.1186/s12896-018-0485-3)
Supplement: Supplementary file 1 — An additional file named Bouvette_Additional_File1.pdf is available that contain Table S1 as well as Figures S1 and S2. Table S1. compares kinetic parameters obtained under conditions that respect or not the steady-state assumption. Figure S1. shows SEC-MALS analysis of purified WT Dicer stored in sucrose/DDM-containing storage buffer for 6 months at − 80 °C. Figure S2. shows preliminary kinetics studies of pre-let-7a-1 cleavage by Dicer under non-steady-state conditions. (PDF 227 kb) [file 12896_2018_485_MOESM1_ESM.pdf]

Supplementary Material

High-yield production of human Dicer by transfection of human HEK293-EBNA1 cells grown in suspension

Jonathan Bouvette[1], Dursun Nizam Korkut[1], Aurélien Fouillen[1,2], Soumiya Amellah[1], Antonio Nanci[1,2], Yves Durocher[1,3], James G. Omichinski[1] and Pascale Legault[1]

[1]Département de Biochimie et Médecine Moléculaire, Université de Montréal, C.P. 6128, Succursale Centre-Ville, Montréal, QC, Canada,
[2]Département de Stomatologie, Université de Montréal, C.P. 6128, Succursale Centre-Ville, Montréal, QC, Canada, H3C 3J7
[3]Human Health Therapeutics Research Centre, National Research Council Canada, Montréal, QC, Canada, H4P 2R2

* correspondence to pascale.legault@umontreal.ca

**Table S1**	**S2**
**Figure S1**	**S3**
**Figure S2**	**S4**

Table S1. Comparison of kinetic parameters obtained under different conditions that respect or not the steady-state assumption.

	Chakravarty et al. [20]	This study (Fig.S2)	This study (Fig.5)
Steady-state assumption	Non-respected	Non-respected	Respected
[Dicer] (nM)	5	5	0.35-3.75
[Substrate] (nM)	25-375	2.5-1000	80-7860
K_M (µM)	0.02-0.03	0.066 ± 0.002	1.2 ± 0.3
k_{cat} (min^{-1})	0.45	0.8 ± 0.1	7.2 ± 0.5
k_{cat}/K_M ($min^{-1}µM^{-1}$)	15-23	12 ± 2	6 ± 1

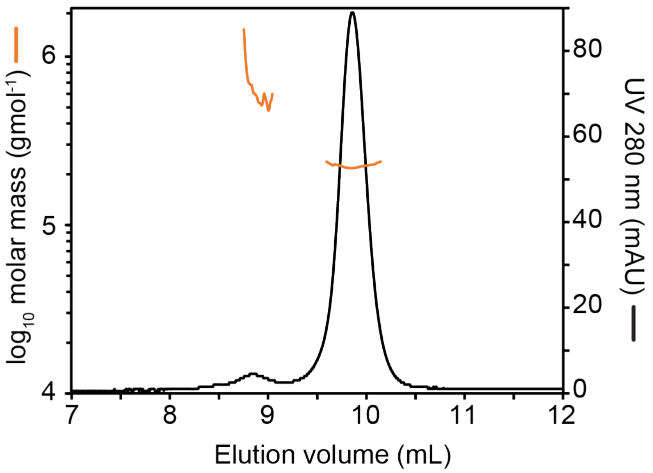

Fig. S1. SEC-MALS analysis of purified WT Dicer stored in sucrose/DDM-containing storage buffer for 6 months at -80˚C. Peak integration of the UV absorbance trace shows that the main peak contains ≥ 94% of the eluted protein. The molar mass calculated from MALS analysis was normalized against BSA to give an average molecular weight of 215 ± 3 kDa with a polydispersity index of 1.00 ± 0.2 over the entire eluted peak. The hydrodynamic radius from quasi-elastic light-scattering (QELS) is 13.7 nm (± 4.5%).

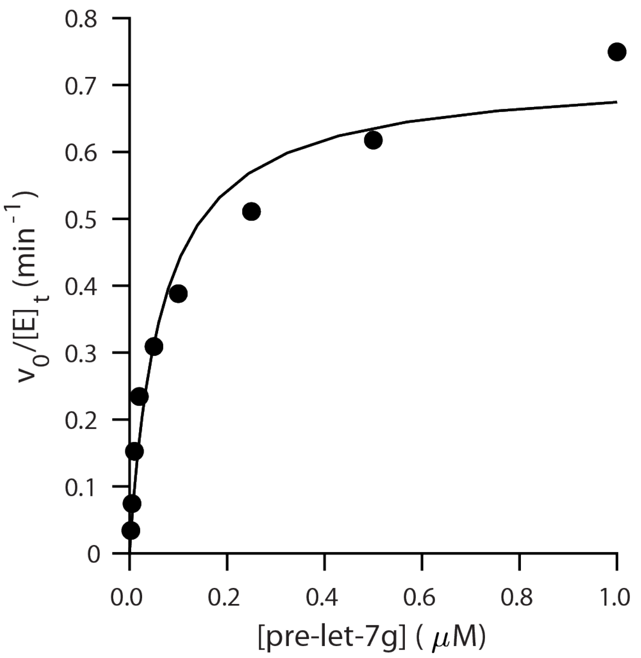

Fig. S2. Preliminary kinetics studies of pre-let-7a-1 cleavage by Dicer under non-steady-state conditions. The turnover frequency (v_0/[E]$_t$) is plotted as a function of substrate concentration (2.5 nM to 1 μM). The kinetic studies and analysis were performed as described in Methods (Dicer cleavage assay), but the concentration of Dicer (5 nM) was kept constant for each substrate concentration. The data shown here were fitted to the Michaelis-Menten equation to provide underestimated k_{cat} (0.72 ± 0.05 min^{-1}) and K_M (65 ± 16 nM) values, since the reactions conditions did not allow for the steady-state assumption (see text). Averages from two independent experiments yield k_{cat} of 0.8 ± 0.1 min^{-1} and K_M of 66 ± 2 nM.
